# Acute Airway Obstruction from Megaoesophagus Secondary to Achalasia Evaluated with Flexible Bronchoscope

**DOI:** 10.1155/2021/8815376

**Published:** 2021-05-08

**Authors:** Jun D. Parker

**Affiliations:** ^1^Department of Anaesthesia, Portland District Health, 141-151 Bentinck Street, Portland, Victoria 3305, Australia; ^2^School of Clinical Medicine, The University of Queensland, St Lucia, Queensland, 4072, Australia

## Abstract

A 94-year-old female presented to the emergency department with acute expiratory stridor. In the absence of an otorhinolaryngologist, an urgent laryngoscopy was performed using a flexible bronchoscope by an anaesthesiologist in the emergency department leading to a change in management. Subsequent radiographs confirmed severe tracheal compression from megaoesophagus secondary to achalasia as the cause of acute airway obstruction. Use of flexible bronchoscope as a diagnostic tool by an anaesthesiologist to evaluate a patient presenting with signs of acute airway obstruction may lead to a safer and more careful airway management planning. Suggestions are also made regarding establishment of emergency surgical airways when conventional approaches fail.

## 1. Introduction

Megaoesophagus secondary to achalasia is a rare cause of acute stridor [1]. Representation with acute stridor after definitive management of achalasia also occurs only rarely [2]. Additionally, oedematous larynx from this condition is extremely uncommon [3]. Moreover, direct visualization of the larynx using a flexible bronchoscope at the first instance in the emergency department in an adult presenting with stridor prior to any other imaging modalities being performed is scarcely reported in the literature. This case report illustrates that the use of flexible bronchoscope in the evaluation of a patient presenting with acute stridor is a valuable tool, especially in a facility without an immediate access to flexible laryngoscope or an otorhinolaryngologist. Suggestions are made regarding establishment of emergency surgical airway when subglottic tracheal compression renders approaches outlined in difficult airway management guidelines difficult or impossible. Written consent was obtained from the patient's medical power of attorney to publish this case report.

## 2. Case Presentation

A 94-year-old female from a high-level care nursing home presented to the emergency department by an ambulance with acute expiratory stridor after being found by the nursing staff with oxygen saturation of approximately 60% on room air. On arrival to the emergency department, her vitals were blood pressure of 180/100 mmHg, respiratory rate of 34 breaths per minute, pulse rate of 90 beats per minute, temperature of 36.3°C, oxygen saturation of 99% on oxygen delivered at 3 litres per minute via nasal prongs, and a Glasgow Coma Scale of 6. The patient had a written not-for-resuscitation order in place.

**Figure 1:**
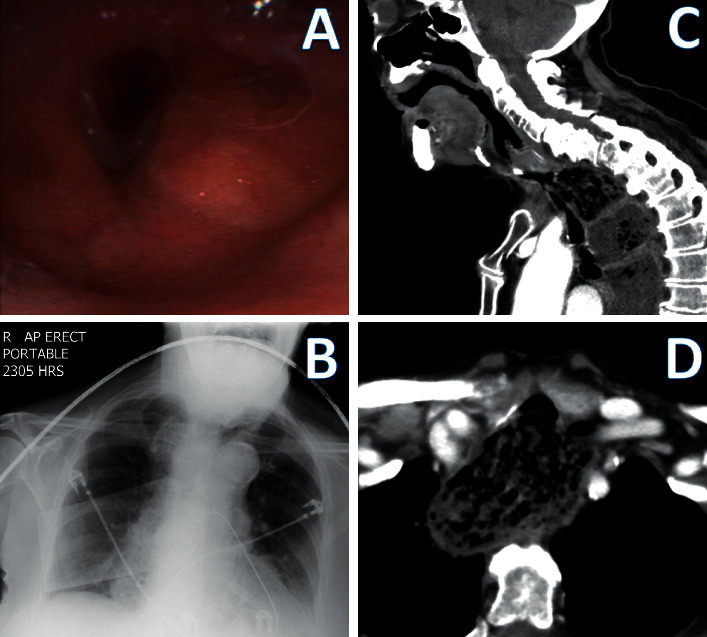
(a) Bronchoscopic image of the laryngeal inlet showing laryngeal oedema. (b) Anterior-posterior view of erect portable chest X-ray. (c) Sagittal view of CT neck showing massively dilated oesophagus and anteriorly displaced trachea and larynx. (d) Axial view of CT neck at the level of clavicles showing massively dilated oesophagus compressing the trachea anteriorly.

On examination, coarse expiratory stridor was maximally heard anterior to the neck and sternum. Venous blood gas showed a pH of 7.08, pCO_2_ of 107 mmHg, pO_2_ of 64 mmHg, and HCO_3_ of 32 mmHg. The sole local surgeon for the hospital was unavailable that evening, and the nearest hospital with an otorhinolaryngologist was located 85 kilometers away. Radiographers were also offsite. As the risk of acute complete airway obstruction was assessed to be imminent, an urgent laryngoscopy was performed using a flexible diagnostic bronchoscope. An Ambu aScope (Ambu A/S, Ballerup, Denmark) Regular Size disposable flexible bronchoscope was passed into the oral cavity by an anaesthesiologist. The laryngoscopy showed laryngeal oedema with narrowed laryngeal inlet with no glottic/supraglottic lesions or foreign bodies (Figure 1). The bronchoscope was not passed into the trachea as sufficient aetiological delineation was deemed to have been achieved, namely, the cause of stridor was likely occurring at the subglottic level secondary to external tracheal compression. Further intervention was assessed to be high-risk and not in the best interest of the patient given the limitations in available clinical support within the facility and the written not-for-resuscitation order. Subsequently, a portable anterior-posterior chest X-ray showed widened mediastinal opacity (Figure 1). Nebulised adrenaline, intravenous dexamethasone 4 mg, and intravenous hydrocortisone 200 mg were administered, and the stridor and respiratory distress gradually disappeared. The patient's medical power of attorney clarified a preference for not transferring the patient to a tertiary facility, and the patient was admitted as an inpatient for observation. It was later revealed that the patient had a previous history of achalasia 2 years ago treated with Botox injection at another facility.

By the next morning, the patient had no audible stridor with return of her baseline cognitive function. A contrast computerized tomography (CT) scan of the neck and chest showed massively dilated oesophagus to the level of cricopharyngeus, compressing the trachea with its narrowest diameter of 5 mm, displacing the trachea and larynx anteriorly (Figures 1 and 1). Unfortunately, she then began to develop intermittent episodes of stridor and delirium. Nasogastric intubation and aspiration were considered, but the risk of iatrogenic oesophageal perforation was assessed to be high on the advice of a gastroenterologist. As her hypoactive delirium worsened, it became clear by day 4 that she was entering into the end-of-life phase and a palliative approach was adopted for her remaining care. She passed away on day 5 of admission.

## 3. Discussion

Megaoesophagus secondary to achalasia is a rare cause of acute stridor [1]. Management strategies include nasogastric tube insertion and aspiration, sublingual glyceryl trinitrate, systemic steroid, nebulized adrenaline, and early endotracheal intubation [1–4]. Successful immediate complete symptomatic relief may be achieved. Clinical consequences, however, may include complete airway obstruction, cardiorespiratory arrest, and sudden death [5]. Early recognition and institution of appropriate management, therefore, are important.

When the laryngeal inlet is restricted, rapid sequence induction and immediate endotracheal intubation may lead to a can't intubate can't oxygenate scenario. Severely compressed and displaced trachea can also affect the conduct of establishing a surgical airway. In these cases, awake fibreoptic intubation can also be difficult or even impossible, and emergency surgical tracheostomy may be required [6]. Immediate assessment of the larynx in the emergency department as demonstrated in this case therefore may assist in a more careful airway management planning.

Evaluation of acute stridor traditionally involved using a flexible laryngoscope to visualize the larynx. Flexible bronchoscopy has been also cited as a valuable tool instead of flexible laryngoscopy. However, the literature on this is limited to paediatrics [7]. Especially in an institution without the availability of flexible laryngoscope, or an otorhinolaryngologist, the use of flexible bronchoscope should be considered as an essential immediate diagnostic tool in evaluating a patient presenting with acute stridor in respiratory distress. However, appropriate care needs to be taken when accessing the glottis.

Difficult airway management guidelines published by anaesthesiology professional bodies universally suggest establishment of an emergency surgical airway as the final step in the can't intubate can't oxygenate scenarios [8]. It includes surgical cricothyrotomy or needle cricothyrotomy approaches [9]. Railroading a size 6.0 mm (internal diameter) oral endotracheal tube over an adult sized bougie is recommended for surgical cricothyrotomy [9], while in needle cricothyrotomy, a conversion to a tracheostomy using a size 5.0 mm (internal diameter) cricothyrotomy kit is recommended [9]. The degree of extrinsic tracheal compression occurring below the level of cricothyroid membrane such as in this case may render insertion of these airway devices difficult or impossible. In the absence of the institutional capability to establish formal surgical tracheostomy emergently, use of a paediatric sized bougie and railroading a smaller oral endotracheal tube or use of a paediatric size cricothyrotomy kit after successful needle cricothyrotomy as recommended in the paediatric anaesthesia literature [10], could be considered as a rescue technique in this situation.

In addition to the findings from the bronchoscope, the presence of expiratory stridor was an important clinical clue to diagnosis. Expiratory stridor indicated that the cause of airway obstruction was intrathoracic, either from tracheobronchial anomalies or external compression of the trachea. Combined with the blood gas findings of severe hypercapnia without hypoxemia, this further supported that the main issue in ventilation was airway obstruction occurring almost exclusively in expiration and not in inspiration. This also explains the prompt restoration of oxygenation from low-flow supplementary oxygen. The likely mechanism behind the blood gas findings therefore involved a combination of reduced tidal volume and air trapping [1].

While achalasia is a rare cause of acute airway obstruction, anaesthesiologists may be called upon as first responders and need to be abreast of its clinical characteristics and develop a safe approach to airway management. Immediate and judicious use of a flexible bronchoscope at the initial assessment can facilitate a more careful and safer airway management planning and may be particularly useful in resource-limited setting such as in rural hospitals where flexible laryngoscopes and otorhinolaryngologists may not be available. Difficult airway guidelines generally focus on immediate establishment of an airway or ventilation. A more diagnostic approach to difficult airway management, however, may be more beneficial under certain circumstances such as this when embarking on airway management according to the difficult airway guidelines may fail and could potentially make an expected difficult airway into a failed airway unsalvageable even with emergency surgical airway techniques.

## Data Availability

The data used to support the findings of this study are available from the corresponding author upon request.

## Abbreviations

CT scan: Computerized tomography scan.
